# Interpreting Expression Data with Metabolic Flux Models: Predicting *Mycobacterium tuberculosis* Mycolic Acid Production

**DOI:** 10.1371/journal.pcbi.1000489

**Published:** 2009-08-28

**Authors:** Caroline Colijn, Aaron Brandes, Jeremy Zucker, Desmond S. Lun, Brian Weiner, Maha R. Farhat, Tan-Yun Cheng, D. Branch Moody, Megan Murray, James E. Galagan

**Affiliations:** 1 Broad Institute of MIT and Harvard, Cambridge, Massachusetts, United States of America; 2 Department of Genetics, Harvard Medical School, Boston, Massachusetts, United States of America; 3 Department of Epidemiology, Harvard School of Public Health, Boston, Massachusetts, United States of America; 4 Department of Pulmonary and Critical Care Medicine, Massachusetts General Hospital, Boston, Massachusetts, United States of America; 5 Department of Engineering Mathematics, University of Bristol, Bristol, United Kingdom; 6 Brigham and Women's Hospital, Harvard Medical School, Boston, Massachusetts, United States of America; 7 Phenomics and Bioinformatics Research Centre, School of Mathematics and Statistics, and Australian Centre for Plant Functional Genomics, University of South Australia, Mawson Lakes, South Australia, Australia; 8 Department of Biomedical Engineering and Department of Microbiology, Boston University, Boston, Massachusetts, United States of America; University of Virginia, United States of America

## Abstract

Metabolism is central to cell physiology, and metabolic disturbances play a role in numerous disease states. Despite its importance, the ability to study metabolism at a global scale using genomic technologies is limited. In principle, complete genome sequences describe the range of metabolic reactions that are possible for an organism, but cannot quantitatively describe the behaviour of these reactions. We present a novel method for modeling metabolic states using whole cell measurements of gene expression. Our method, which we call E-Flux (as a combination of flux and expression), extends the technique of Flux Balance Analysis by modeling maximum flux constraints as a function of measured gene expression. In contrast to previous methods for metabolically interpreting gene expression data, E-Flux utilizes a model of the underlying metabolic network to directly predict changes in metabolic flux capacity. We applied E-Flux to *Mycobacterium tuberculosis*, the bacterium that causes tuberculosis (TB). Key components of mycobacterial cell walls are mycolic acids which are targets for several first-line TB drugs. We used E-Flux to predict the impact of 75 different drugs, drug combinations, and nutrient conditions on mycolic acid biosynthesis capacity in *M. tuberculosis*, using a public compendium of over 400 expression arrays. We tested our method using a model of mycolic acid biosynthesis as well as on a genome-scale model of *M. tuberculosis* metabolism. Our method correctly predicts seven of the eight known fatty acid inhibitors in this compendium and makes accurate predictions regarding the specificity of these compounds for fatty acid biosynthesis. Our method also predicts a number of additional potential modulators of TB mycolic acid biosynthesis. E-Flux thus provides a promising new approach for algorithmically predicting metabolic state from gene expression data.

## Author Summary

The ability of cells to survive and grow depends on their ability to metabolize nutrients and create products vital for cell function. This is done through a complex network of reactions controlled by many genes. Changes in cellular metabolism play a role in a wide variety of diseases. However, despite the availability of genome sequences and of genome-scale expression data, which give information about which genes are present and how active they are, our ability to use these data to understand changes in cellular metabolism has been limited. We present a new approach to this problem, linking gene expression data with models of cellular metabolism. We apply the method to predict the effects of drugs and agents on *Mycobacterium tuberculosis (M. tb)*. Virulence, growth in human hosts, and drug resistance are all related to changes in *M. tb*'s metabolism. We predict the effects of a variety of conditions on the production of mycolic acids, essential cell wall components. Our method successfully identifies seven of the eight known mycolic acid inhibitors in a compendium of 235 conditions, and identifies the top anti-TB drugs in this dataset. We anticipate that the method will have a range of applications in metabolic engineering, the characterization of disease states, and drug discovery.

## Introduction

Metabolism is central to cell physiology and metabolic disturbances play a role in numerous disease states. Despite its importance, the ability to study metabolism at a global scale using genomic technologies is limited. In principle, complete genome sequences describe the range of metabolic reactions that are possible for an organism, but cannot quantitatively describe the behaviour of these reactions. Gene expression data provide global insight into the regulation of metabolic reactions, but methods for inferring the behaviour of metabolic networks, and particularly metabolic flux, from these data are limited. There is thus a need to develop computational approaches that utilize available genomic data to make inferences about metabolism at the level of large scale metabolic networks.

One approach to computationally studying metabolism is to develop detailed models based on coupled differential equations describing the dynamics of enzyme action. Such models, however, require measuring numerous kinetic parameters that can be prohibitively difficult for large systems and for organisms – such as infectious disease agents – that are difficult to work with experimentally.

Flux balance analysis (FBA) is an alternative approach to modeling metabolism without developing detailed simulation models that include enzyme kinetics [1]–[4]. It exploits the fact that the stoichiometries of metabolic reactions are not organism-dependent but are fixed by chemistry and mass balance. Moreover, the availability of complete genome sequences is enabling the reconstruction of metabolic networks whose constituent reactions have known stoichiometries. Flux balance analysis also exploits the fact that enzyme dynamics occur quickly compared, for example, to regulatory changes in gene expression: when the relevant laboratory time period (often hours) is much longer than the chemical reaction times (typically minutes), transient dynamics last for only a small portion of time period considered, after which the metabolic network functions at steady state. FBA is a method for utilizing universal reaction stoichiometries to predict a network's capability to produce a metabolic objective under steady-state conditions.

Briefly, FBA represents a metabolic network by capturing the stoichiometries of constituent reactions in a stoichiometric matrix, S, and describing a flux configuration as a set of rates at which the reactions in a network are moving (ie the set of reaction fluxes). FBA requires that constraints for some reactions be known, reflecting their maximum or minimum rates. These constraints can either be measured (e.g. uptake reactions) or calculated from physical parameters (e.g. oxygen diffusion) or thermodynamic constraints. In many cases, the constraints can be related to the degree of enzymatic activity for the given reaction. The matrix S and the set of reaction constraints define the set of all possible flux configurations at steady state. A flux configuration can be visualized as a vector in flux space, and all flux configurations that are feasible at steady state lie within a cone in this space, called the flux cone. The core approach of FBA is to choose a metabolic objective which is a linear function of fluxes, and then use linear programming to optimize this objective subject to the constraints. The algorithm results in one or more flux configurations that are optimal for the chosen metabolic goal, and the optimal production capacity of that objective.

FBA provides a method for exploring capabilities and states of a metabolic system at steady state, and genome scale metabolic models can be reconstructed based on annotated genome sequences coupled with literature curation [1],[2]. FBA has been used to successfully predict the metabolic phenotype of gene knockouts [1]–[3], and the use of metabolic modeling in this case has the advantage of predicting nutrient-dependent phenotypes. FBA has also been used to predict the time courses of growth, substrate uptake, and metabolite production by both *Escherichia coli* and *Mycobacterium tuberculosis* using a pseudo-steady-state dynamic modeling approach [4]–[6]. FBA has recently been used as part of an integrated analysis scheme for drug identification; there is a recent publication (targetTB) by Raman et al. that reports this approach [7].

While powerful, FBA is limited in that it does not take into account the gene regulatory state, as described for example by gene expression data. In effect, the basic approach predicts metabolic capabilities assuming all reactions have the same maximum capacity. Indeed, many of the errors in the prediction of gene knockout phenotype were traced back to the lack of gene regulation in standard FBA models [1],[2]. Incorporating a Boolean model of gene regulation with FBA allows the prediction of more biologically realistic dynamic behaviour, including for example a diauxic shift in response to changing carbon source availability [8]. However, this approach reduces gene expression to Boolean variables, using either a constant value or 0 for the upper flux bound, rather than making use of direct measurements of gene regulation through whole cell expression data.

We have developed a method, which we call “E-Flux”, to predict metabolic capacity based on expression data. E-Flux extends FBA by incorporating gene expression data into the metabolic flux constraints. We applied E-Flux to *M. tuberculosis* (*M. tb*), the pathogen that causes tuberculosis (TB). An estimated one third of the world's population has been exposed to this disease, which is estimated to kill 1.6–1.8 million annually worldwide. Multiple drug resistant (MDR) and extensively drug resistant (XDR) strains of tuberculosis are emerging worldwide, so the development of new drugs is of the essence. Bacterial metabolism plays an important role in TB pathology, both in terms of metabolic alterations associated with intracellular growth [9]–[12] as well as through the production of metabolic products associated with virulence – including mycolic acids [13]–[15]. Given *M. tb*'s slow growth rate, the hazards of experimenting directly with this infectious organism, and limitations in measuring all metabolites simultaneously, there is considerable motivation to augment experimental approaches with computational methods for predicting *M. tb* metabolism.

We used E-Flux to predict the impact of drugs and environmental conditions on mycolic acid biosynthesis capacity in *M. tb*, based on a compendium of expression measurements from these conditions. Our method successfully identifies seven of the eight known inhibitors of mycolic acid or fatty acid production that were present in the compendium. E-Flux also correctly predicts whether conditions are directly inhibiting mycolic acid production, or inhibiting production indirectly through other mechanisms. Our method thus provides a promising approach to modeling metabolic state from whole cell measurements of gene regulation.

## Results

### Method Overview

The key innovation underlying the E-Flux approach is that we use expression data to model the maximum possible flux through metabolic reactions. When the expression for a particular enzyme-coding gene is low (relative to some reference), we place a tight constraint on the maximum flux through the corresponding reaction(s). When expression is high we place a looser constraint on the flux through the reaction(s). We then use FBA with the applied constraints and an appropriate objective function to determine a corresponding metabolic state or optimal metabolic capacity.

**Figure 1:**
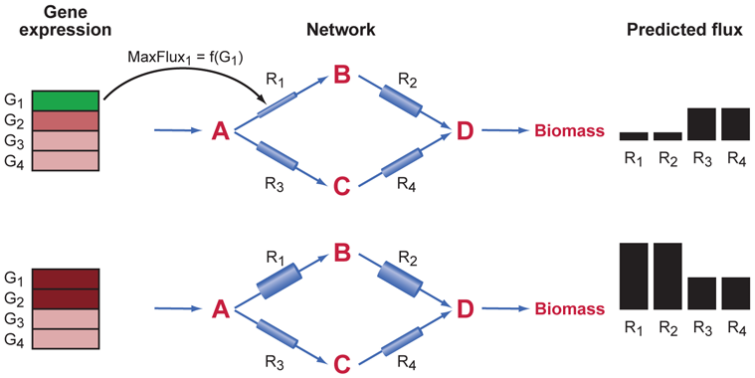
Illustration of E-Flux method. The core idea of the method is that we use gene expression to set maximum flux constraints on individual reactions. This can be illustrated as pipes of different widths around each reaction. Here we show a simple model comprised of 4 metabolites (A–D), 4 internal reactions, an uptake reaction for A, and a reaction converting D to biomass. On the left are simulated gene expression data for 4 genes whose enzymes catalyze the 4 internal reactions (green – lower expression, red – higher expression). In the top panel, G1 is poorly expressed; this can be conceptualized as a thin pipe around reaction 1 as shown. In the bottom panel, G1 and G2 are highly expressed, corresponding to a wider pipe for these reactions. Under conditions in which uptake of A is not limiting, we would predict more flux through R1 and R2 in the bottom panel relative to the top panel and R3 and R4. This is shown by the bars on the right. The specific conditions giving rise to the qualitative predictions on the right are given in the Methods section.

Conceptually, our method can be understood as setting the width of “pipes” around particular reactions as a function of expression state. Figure 1 illustrates this for a simple metabolic model with 4 metabolites and 4 internal reactions catalyzed by enzymes corresponding to 4 genes, together with an uptake reaction and a reaction converting one metabolite into biomass. Two different sets of simulated gene expression data are shown in the two panels. In the top panel, G_1_ is poorly expressed. Our method models this conceptually as a thin pipe around reaction 1, limiting the maximum flux through this reaction. Conversely, in the bottom panel, G_1_ is highly expressed corresponding to more possible flux (a wider pipe). Under conditions in which substrate is not limited, we would predict more flux through reactions 3 and 4, and less through reactions 1 and 2 in the top panel. Conversely, we might predict more flux through reactions 1 and 2, and less through 3 and 4, in the bottom panel.

Geometrically, setting maximum flux constraints according to gene expression reshapes the flux cone. Different gene expression states result in different flux cone geometries, which can lead to different solutions for the same metabolic objective. Reshaping the flux cone and thus generating different flux configurations is similar to the approach used to predict phenotypes from gene knockouts using FBA [1]–[3] and for coupling Boolean regulatory models with metabolic models [8]. However, such approaches have used constraints that are the same for all reactions except those that are turned off. By contrast, E-Flux shapes the cone not by turning individual genes on or off, but by giving many or all genes in the model a range of possible flux limits. More importantly, we are reshaping the flux cone on the basis of empirical measurements of gene expression.

Our method does not assume that enzyme concentrations, enzyme activities, or realized reaction fluxes are determined by mRNA expression values. Indeed, the true flux for a reaction depends on the enzyme kinetics and concentration, as well as the concentration of metabolites. The effective enzyme concentration in turn depends on gene expression, transcription and translation, post-translational modification and degradation. It would be prohibitive to determine these values for many reactions in an organism.

However, the biological rationale behind our method is that expression data provide measurements on the level of mRNA for each gene. If there were limited accumulation of enzyme over the time course considered, and given a particular level of translational efficiency, the level of mRNA can be used as an approximate upper bound on the maximum available protein and hence as an upper bound on reaction rates to some level of approximation. This allows us to extend flux balance analysis from an algorithm that assumes that all reactions have the same constraint, as has been done previously, to an approach making use of condition-dependent, empirical data. E-Flux allows us to link such data directly to changes in metabolic capability. We discuss rationale behind our method further in the Discussion.

Mathematically, our approach modifies FBA as follows. FBA involves solving the following optimization problem:
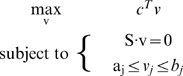
(1)where v is a flux vector representing a particular flux configuration, S is the stoichiometric matrix, c is a vector of coefficients that defines a linear objective function c^T^v, and a_j_ and b_j_ are the minimum and maximum fluxes through reaction j. We assume that we have a set of expression measurements for some or all of the genes associated with the reactions in S.

The core E-Flux method chooses the maximum flux, b_j_, for the j^th^ reaction according to a function of the expression of gene j and associated genes:
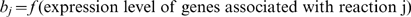
(2)


If the reaction catalyzed by the corresponding enzyme is reversible then a_j_ = −b_j_, otherwise a_j_ = 0. For the results presented here, “associated genes” refers both to genes that are components of the same enzyme complex, and genes associated with separate isozymes of the reaction. In the latter case, we choose *f* to be a monotonically increasing function of the expression of the corresponding genes. In general b_j_ can also depend on genes that modulate the activity of the enzyme for reaction j and *f* can thus take on a more general form. In the Discussion section, we examine the question of which genes to associate with a particular maximum flux constraint and the functional form of *f*.

### Application of E-Flux to *M. tuberculosis* Mycolic Acid Biosynthesis

We tested E-Flux on two metabolic models that include the biosynthesis of mycolic acids in *M. tb*. The first model consisted of just those reactions underlying mycolic acid production. Mycolic acids are cell wall components characteristic of mycobacteria and essential for the survival of the bacterium [13]. Because the mycolic acid biosynthetic pathway does not exist in non-actinmycetales species, including humans, it is the target of several of the most common antibiotics used to treat TB including isoniazid, thiolactoymycin and ethionamide. Moreover, a metabolic sub-model for this pathway has been previously published [7]. This model, which included 28 proteins, 219 reactions and 197 metabolites, contained four sub-pathways representing the production of malonyl CoA, the fatty acid synthase (FAS) I and II pathways, and the condensations of the resulting FAS products into alpha-, methoxy- and keto- mycolic acids. We augmented this model with two additional genes subsequently identified with mycolic acid biosynthesis [16].

We analyzed the microarray data from the Boshoff TB gene expression compendium [17]. This compendium consists of data from several studies, totalling 437 microarray experiments which measured the transcriptional response of *M. tuberculosis* to 75 different substances and conditions, including known anti-tubercular drugs, growth conditions and unknown compounds. Specifically, this set also included eight known inhibitors of mycolic acid production. Our goal was to use E-Flux to predict the impact of each of these compounds or conditions on mycolic acid biosynthetic production in *M. tuberculosis*.

To explore the method's relevance to data from diverse groups and for a variety of experimental conditions, we also analyzed a set of expression data of Karakousis et al. [18]. These authors analyzed global gene expression profiles to study the action of isoniazid, a mycolic acid inhibitor and front-line antitubercular agent, on several models of *M. tb*'s dormancy phase.

### Application to Genome Scale *M. tuberculosis* Metabolic Model

Two genome-scale metabolic models are available for M. tuberculosis, namely those of Beste et al. [6] and Jamshidi and Palsson [19]. To validate that our method scales to genome-wide metabolic model, we applied E-Flux to the comprehensive model of *M. tuberculosis* metabolism of Beste et al. [6]. This was chosen because the model contains more genes and the predictions for gene essentiality were better than those of Jamshidi and Palsson, whose focus was more on growth rates. Since our analysis is comparative in nature we felt that the qualitative advantage of a model with more correct gene essentiality was relevant.

Beste et al.'s model [6] was modified by merging this genome scale model with the mycolic acid submodel of Raman et al. [7]. Specifically, we removed mycolic acid reactions from the genome-scale model and replaced them with the mycolic acid reactions in Raman et al.'s model, and normalized the bounds on exchange reactions (see Methods and Supplementary Material for more details). The net result was to replace Beste et al.'s representation of mycolic acids with that of Raman et al., as the latter is more detailed and as this allows direct comparison of the results of E-Flux in the two models.

As with the model of mycolic acid production, we applied E-Flux to the genome scale model to predict the impact of each of the compounds or conditions in the Boshoff TB gene expression compendium on mycolic acid biosynthetic production [17].

### Computational Approach

**Figure 2:**
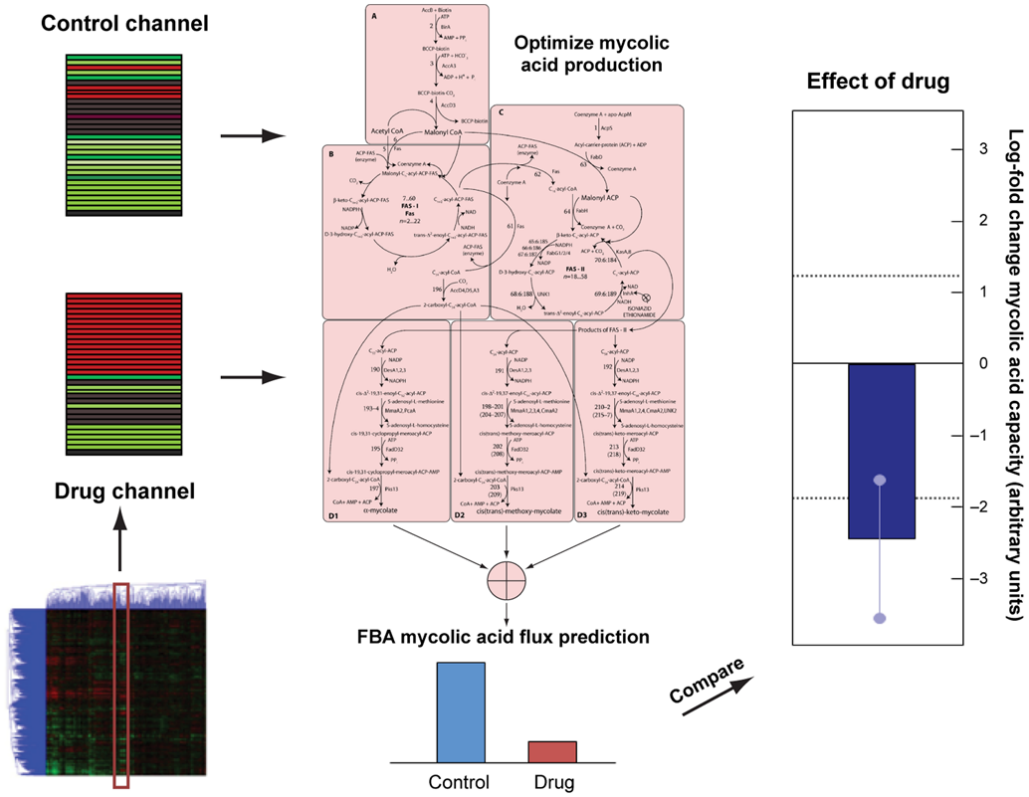
Applying E-Flux to mycolic acid biosynthesis. For each experiment from [17], we separated the corresponding drug or condition (cy5) and control channels (cy3). When first applied expression data from the control channel to set constraints in the mycolic acid model (the model schematic is adapted from [7]) and used FBA to predict maximum mycolic acid biosynthetic capacity (bottom light blue bar). We then predicted maximum mycolic acid flux for the drug by applying expression from this channel (bottom red bar). We compared both predictions to assess the relative impact of the drug or condition on mycolic acid biosynthetic capacity (right blue bar). The dotted lines on the right indicate 95% confidence intervals for differences that would be expected from comparing two control channels. The barbells represent condition specific error bars. A similar method was used for the genome scale model (Methods).

Our computational approach is shown in Figure 2. We first pre-processed the expression data using a previously published analysis of variance (ANOVA) method [20]. This method utilizes replicates within and between conditions to estimate sources of noise including variations between binding affinities at different spots on each chip, variations from chip to chip, various binding affinities from gene to gene, dye effects, and biological variation within replicates. We also performed the method using data pre-processed with a median-adjustment to the control channel median of each chip. Our predictions were not substantially altered by the choice of pre-processing.

Following pre-processing, we separated the drug or condition (cy5) and control channels (cy3). For each experiment we first applied expression data from the control channel to set constraints on maximum fluxes of reactions in the model. We then used FBA to find a flux configuration that maximized overall mycolic acid biosynthesis (bottom light blue bar in Figure 2). Similarly, we predicted maximum mycolic acid production for the corresponding drug condition by applying expression from this channel (bottom red bar). We compared both predictions to assess the relative impact of the drug or condition on mycolic acid biosynthetic capacity (right blue bar). In the case of Figure 2, we would predict that the drug inhibits mycolic acid production.

To perform FBA for the mycolic acid biosynthetic model, we utilized an objective function representing total mycolic acid production. This model does not suggest that *M. tb* is in fact trying to maximize production of mycolic acids, but this objective function allows us predict the maximum amount of mycolic acid the model could produce under the given constraints. For the genome-scale model, we used the same objective function. We were also able to use the biomass objective as given in [6] (Methods).

**Figure 3:**
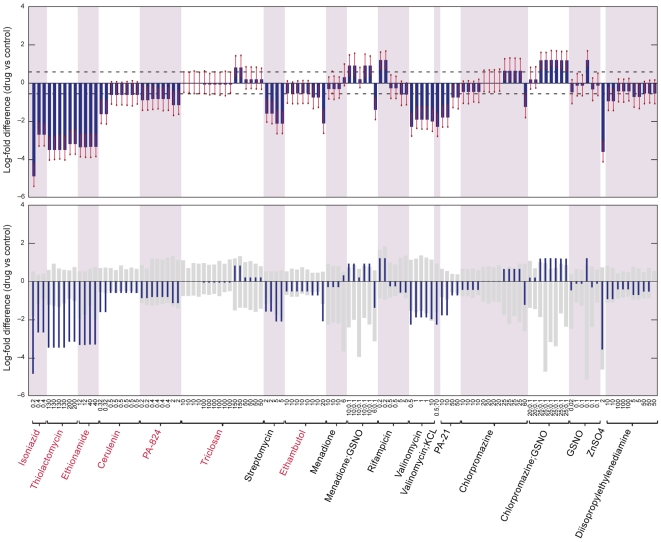
Selected predictions of E-Flux applied to mycolic acid biosynthesis in M. tuberculosis. Top panel: predictions and prediction significance are displayed for each condition as described in Figure 2. Conditions are arranged on the x-axis as indicated by the labels. Many conditions are replicates of the same compound, possibly with different concentrations. Replicates are indicated by the background shading and brackets in the horizontal axis label. Numbers in the brackets are concentrations for each replicate. Bottom panel: the specificity of each prediction for mycolic acid biosynthesis is displayed. The dark bars indicate the prediction strength as in the top panel. The light grey bars indicate the 95% confidence interval for predictions made by randomizing gene labels. Dark bars smaller than light bars indicate non-specific predictions. Known fatty acid inhibitors are shown as red on the horizontal axis label.

Differences in predicted mycolic acid flux arising from comparing the drug and control channel could arise due to fluctuations in gene expression measurements independent of drug effects. To determine whether a particular difference could be explained by such fluctuations, we resampled data on the control channels with noise fluctuations derived from the ANOVA analysis to understand how much variation in the predictions would result from comparing two different control channels. The 95% confidence interval for predictions from resampled control channel data is represented as the dotted lines in Figure 3 and is the same for all experiments. To generate error bars for each prediction we resampled both control and drug channels with noise drawn from this distribution; the resulting error bars are shown in Figure 3.

We also wished to determine if predicted differences were specific to mycolic acid biosynthesis or reflected a more general change in metabolism. For example, a predicted inhibition of mycolic acid production might be due to an overall suppression of gene expression or metabolism. To this end, we randomly relabelled genes within each data set and found predictions using E-Flux. Repeating this permutation and computation multiple times, we calculated a null distribution associated with non-specific effects on mycolic acid production for each condition. The 95% ranges for these distributions are shown as grey bars in Figure 3.

### Predicted Mycolic Acid Biosynthesis Modulators

We applied the approach shown in Figure 2 to all 437 experiments in the Boshoff data set. The results for the mycolic acid biosynthetic model are summarized in Tables 1 and 2 and details for a subset of predictions are shown in Figure 3. The most noteworthy aspect of these results is that of the eight known inhibitors of mycolic acid tested in the Boshoff data set, E-Flux correctly predicts seven. More generally E-Flux identifies as modulators all of the drugs used against tuberculosis that are known to affect the mycolic acid pathway.

**Table 1:** Summary of E-Flux predicted mycolic acid inhibitors from Boshoff data set.

Predicted Inhibitors			
Isoniazid	Specific	Strong	Known
Thiolactomycin	Specific	Strong	Known
Ethionamide	Specific	Strong	Known
ZnSO4	Non-specific	Strong	New
Ethambutol	Specific‡	Weak‡	Known
Cerulenin	Specific‡	Weak‡	Known
PA-21	Specific‡	Weak	New
Streptomycin	Non-specific	Weak	New
Valinomycin	Non-specific	Weak	New
Amikacin	Non-specific	Weak‡	New
Pyrazinamide * ‡	Non-specific	Weak	Known
Tetracycline	Non-specific	Weak	New
Dubos-NRP1; Dubos-NRP1+KNO3	Non-specific	Weak*	New
PA-824	Non-specific	Very weak	Known
Chlorpromazine‡	Non-specific	Very weak	New
Capreomycin	Non-specific	Very weak	New
Synthetic pyridoacridine analog (124196)‡	Specific	Very weak	New
Ascedidemin (111895)‡	Non-specific	Very weak	New
Rifapentine‡	Non-specific	Very weak	New
Procept 6776, 6778‡	Non-specific	Very weak	New
Succinate, palmitate in minimal medium*	Non-specific	Very weak	New
Starvation conditions	Non-specific	Very weak	Expected

Results are shown for all significant predictions from the set of 437 experiments in Boshoff et al [17]. Of the *seven* known inhibitors of fatty acid biosynthesis, E-Flux correctly predicts 6. Strong effects are defined as effects showing greater than +/−3 log change between control and drug; weak inhibitory effects have inhibition less than −1.5 log change and very weak effects less than −1. Weak enhancing effects have greater than +1 log change. Specific effects indicate effects on mycolic acid biosynthesis as contrasted to effects over a broad range of pathways – see text for more details.

* Prediction made only for certain replicates.

‡ Prediction made only for certain doses.

Starvation conditions were phosphate- or Tris-buffered saline containing 0.05% Tween 80 (PBST or TBST) [17]. Conditions with both enhancing and inhibiting predictions among replicates were excluded.

**Table 2:** Summary of E-Flux predicted mycolic acid enhancers from Boshoff data set.

Predicted Enhancers			
Chlorpromazine/GSNO	Non-specific	Weak*	New
Rifapentine‡	Non-specific	Weak	New
Rifampicin‡	Non-specific	Weak	New
Chlofazimine/GSNO	Non-specific	Weak	New
GSNO	Specific	Strong*	New
Menadione/GSNO	Non-specific	Very weak*	New
Triclosan	Non-specific	Very weak*	Incorrect?

Results are shown for all significant predictions from the set of 437 experiments in Boshoff et al [17]. Strong effects are defined as effects showing greater than +/−3 log change between control and drug; weak enhancing effects have greater than +1 log change, and we list two very weak effects defined as prediction lying outside the 95% null confidence interval. Specific effects indicate effects on mycolic acid biosynthesis as contrasted to effects over a broad range of pathways – see text for more details.

* Prediction made only for certain replicates.

‡ Prediction made only for certain doses.

Conditions with both enhancing and inhibiting predictions among replicates were excluded.

The application of E-Flux to the the *M. tb* genome-scale model produced an identical set of predicted inhibitors and enhancers with the same specificity and predicted strength as those in Table 1 and Table 2 although the quantitative predictions differed slightly (Supplementary Material). This was true regardless of whether we used the mycolic acid objective function from [7] or the biomass objective function of [6]. Our method is thus applicable to the both targeted metabolic models as well as genome-scale metabolic reconstructions.

The strongest predicted inhibitors included isoniazid (INH) and ethionamide. Isoniazid, a first-line drug for TB, is a prodrug that is activated by the bacterial catalase-peroxidase enzyme KatG [21]. Activation leads to the development of INH-NAD and INH-NADP adducts that inhibit InhA and FabG1 respectively [22],[23]. Both InhA and FabG1 are components FAS-II cycle of mycolic acid biosynthesis [22],[24]. Ethionamide is a structural analog of INH and is thought to also target InhA [25]. Both isoniazid and ethionamide are predicted as strong selective inhibitors of mycolic acid biosynthesis.

E-Flux also predicts thiolactomycin and ethambutol as strong selective inhibitors. Thiolactomycin is a natural product produced by both *Nocardia* and *Streptomyces* and is a potent and highly selective inhibitor of the type II dissociated fatty acid synthase of plants and bacteria [26]. Ethambutol inhibits the arabinosyl transferases in the synthesis of arabinogalactan, and prevents the attachment of mycolic acids to the 5′-hydroxyl groups of D-arabinose residues of arabinogalactan thus obstructing the formation of the mycobacterial cell well [27]. Interestingly, E-Flux only predicted inhibition for the highest concentration of ethambutol. If correct, this mechanism may lead to reduced quantities of mycolate polymer, with subsequent build-up of free mycolate, and it seems possible that mycolic acid biosynthesis would be down-regulated in response.

In addition, E-Flux predicts a number of weaker inhibitors, including two drugs known to impact mycolic acid. Cerulenin is a fungal mycotoxin that is known to inhibit both FAS-I and FAS-II cycles in mycolic acid synthesis in *M. tb* [28]. Pyrazinamide is a pro-drug of pyrazinoic acid, and inhibits the FAS1 pathway of mycolic acid synthesis in M. *tb* [29]. PA-824 is a newer nitroimidazopyran drug currently in clinical trials [30]. PA-824 inhibits both lipid and protein synthesis by as yet unknown mechanisms. E-Flux predicts that PA-824 inhibits mycolic acid synthesis at the higher concentration replicates but not at lower ones. PA-824 was not predicted as a specific inhibitor of mycolic acid biosynthesis, although this may be due to the additional effect of PA-824 on protein synthesis genes or to the relative weakness of the predicted mycolic acid inhibition.

E-Flux also predicts several novel compounds not previously associated with inhibition of mycolic acid biosynthesis. These include predictions of weak effects for the protein synthesis inhibitor streptomycin and the ionophore valinomycin. These compounds are predicted as non-specific inhibitors consistent with an overall impact on metabolism. PA-21 was also predicted to be a weak and marginally specific inhibitor, although the mechanism of this compound has not been reported. Of the novel predicted inhibitors, only ZnSO4 was predicted to have a strong effect with marginal specificity. However, only a single replicate for ZnSO4 is present in the Boshoff data set, and preliminary experimental data suggest that this prediction is likely a false positive.

Interestingly, E-Flux also predicted a small number of compounds that may enhance fatty acid biosynthesis. Menadione and chlorpromazine are predicted to be weak non-specific enhancers, although these results are convolved with GSNO and one instance of GSNO in isolation was predicted as a strong and specific enhancer. However, it is noteworthy that menadione has been reported to increase fatty acid production in human fat cells, in addition to a number of other metabolic effects [31]. Chlorpromazine is a phenothiazine, a class of compounds recently proposed as possible drugs targeting multi-drug resistant tuberculosis. GSNO is a nitric oxide donor toxic to mycobacteria [32] whose mechanism of antimycobacterial action of GSNO is unknown [33],[34]. Extracellular glutathione is converted to a dipeptide, which is transported into the bacterial cells by the multicomponent ABC transporter dipeptide permease [32].

Curiously, triclosan was also predicted as an enhancer. Triclosan is known to inhibit the enoyl-ACP reductase of FASII [35]. Although we predict no significant effect at low concentrations, E-Flux predicts a significant upregulation of mycolic acid production at the highest concentration. It has been observed that triclosan acts through more than one mechanism [35] and may lead to upregulation of fatty acid metabolism

**Figure 4:**
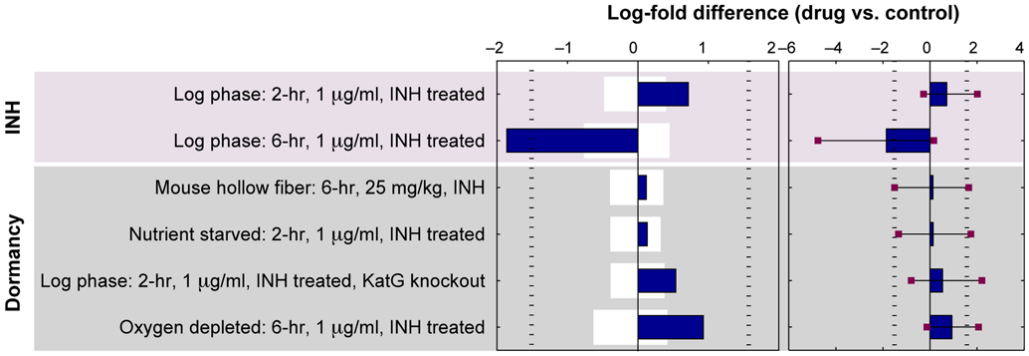
Predictions of E-Flux applied to data on *M. tb*'s response to isoniazid under dormancy conditions. Top panel: predictions and prediction significance are displayed for each condition as described in Figure 2. Isoniazid shows a significant effect after 6 hours but shows no comparable inhibition under dormancy conditions. Note that the lack of inhibitory effect shown at 2 hours may reflect incompleteness of the transcriptional at this time, in which case the most relevant comparison is to the oxygen depletion condition (6 hr) showing no significant inhibition for this dormany model. Bottom panel: the specificity of each prediction for mycolic acid biosynthesis is displayed. The dark bars indicate the prediction strength as in the top panel. The light grey bars indicate the 95% confidence interval for predictions made by randomizing gene labels.

The data of Karakousis et al. [18] on the transcriptional response of dormant *M. tb* to isoniazid provide the opportunity to examine the predictions of E-Flux for dormant tuberculosis. Though it is a strong inhibitor of mycolic acid biosynthesis, isoniazid has little activity against *M. tb* under oxygen deprivation or nutrient starvation [36]. Consistent with this, Karakousis et al. [18] found that the transcriptional signature associated with isoniazid's activity in non-dormant tuberculosis was abolished under conditions of dormancy. The results of E-Flux applied to these data are shown in Figure 4. E-Flux correctly shows a strong and significant inhibition of mycolic acid biosynthesis after 6 hours, but shows no effect of isoniazid for any of the four dormancy models in the dataset. This not only confirms the result for isoniazid from the Boshoff compendium [17] but provides an indication that E-Flux may be a useful tool in analyzing expression profiles for dormant *M. tb* under a range of treatment conditions, when such data become available.

### Comparison to Gene Expression Clustering

**Figure 5:**
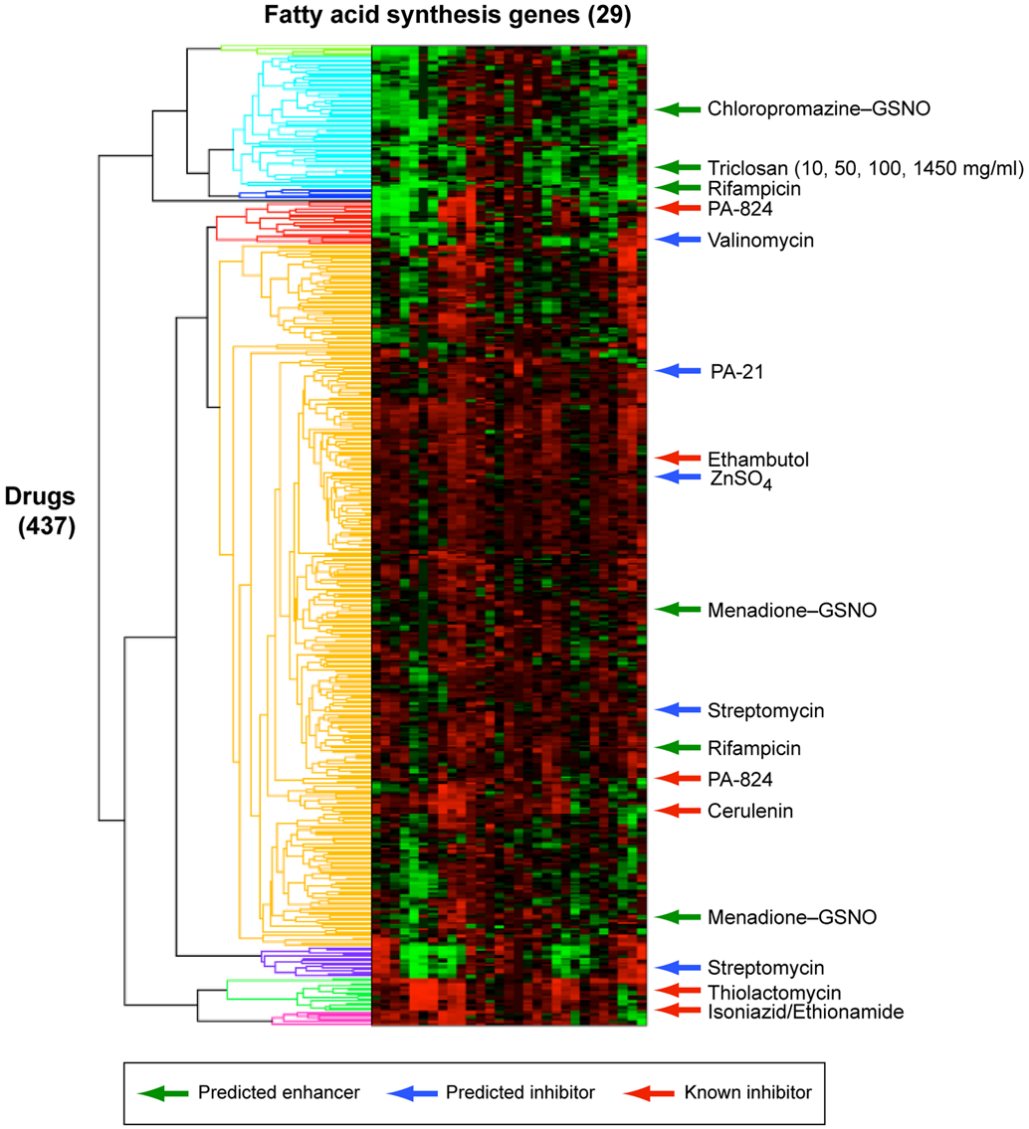
Clustering of experiments in the Boshoff compendium using expression of the mycolic acid biosynthetic genes. Clustering was performed with hierarchical clustering using pearson correlation for the distance metric and the average linkage method. Known mycolic acid inhibitors do not cluster based solely on gene expression. This is consistent with the clustering of all *M. tb* genes reported by [17]. Similarly, predicted inhibitors and enhancers do not form distinct clusters.

To rule out that our predictions reflect similarities in gene expression independent of metabolic modeling, we clustered the expression of the 29 genes used in the mycolic acid biosynthetic model across all 437 experiments in the Boshoff data set. As can be seen in Figure 5, known inhibitors do not form a single cluster. This is consistent with the results of clustering all *M. tb* genes as reported by Boshoff et al. [17]. Similarly, inhibitors and enhancers predicted by E-Flux also do not form a single cluster. Furthermore, predicted inhibitors do not obviously fall into clusters with previously known inhibitors, suggesting that using a metabolic model allows the discovery of distinct routes to inhibition or enhancement of an objective, beyond similarity of gene expression with known inhibitors. More fundamentally, in contrast to supervised classification methods, E-Flux does not require data from compounds with a known effect to calibrate the method (i.e. an initial training set is not required). In particular, no mycolic acid enhancers are currently known, and thus a method designed to classify new enhancers by comparing expression profiles to known compounds would not be applicable. We consider the differences between E-Flux and expression classification in more detail in the Discussion section.

## Discussion

In this paper, we have presented a novel method for predicting metabolic capacity from gene expression data. E-Flux extends flux balance analysis to predict characteristics of steady state metabolism that correspond to specific empirically-measured gene expression states. The key innovation of our method is that we use gene expression data to model the maximum flux through individual metabolic reactions.

We have used E-Flux to predict the impact of drugs, drug combinations, and environmental conditions on mycolic acid biosynthetic capacity in *M. tuberculosis*, based on microarray data from the Boshoff TB expression compendium. E-Flux correctly predicts seven of eight inhibitors of mycolic acid biosynthesis within this compendium, and correctly predicts the specificity of this inhibition for mycolic acid biosynthesis in all but one case. E-Flux also predicts a small number of additional potential inhibitors, as well as potential enhancers of mycolic acid production. We also tested E-Flux on data for dormant *M. tb* treated with isoniazid, and it correctly predicts the difference in effect of this agent in several dormancy models.

E-Flux thus provides a potentially powerful tool for exploring metabolic state (which is relatively difficult to measure) from gene expression state (which is relatively simple to measure in many circumstances). This is particularly significant for tuberculosis given the difficulty of working with *M. tb*, the availability of many microarray experiments for this organism (www.tdbd.org), and the essential role of metabolism in the pathogenesis of *M. tb*.

### Gene Expression and Maximum Flux Constraints

The key principle underlying the E-Flux method is that mRNA levels for enzymes approximate an *upper bound* on the potential flux through the corresponding metabolic reactions, i.e. for a particular level of translation and degradation, the amount of mRNA sets an upper bound on the amount of available enzyme. The amount of available enzyme is in turn proportional to maximum flux (e.g. Vmax) through a particular reaction.

We acknowledge that mRNA expression is not sufficient to determine fluxes or, in many cases, true upper bounds on fluxes, but nonetheless argue that including mRNA expression data into flux balance models provides a new and useful way to connect expression data with models cellular metabolism, and is an improvement upon effectively assuming that all reactions that are present have the same maximum flux. The degree of correlation between mRNA and protein levels is an area of ongoing research [37]. There are conflicting reports regarding the correlation between mRNA and protein levels [38], but some whole genome studies have reported modest correlations. A study of 289 proteins in *Saccharomyces cerevisiae*, for example, reported a correlation of 0.61 after correcting for methodological issues [39]. Correcting for methodological noise and potential non-linearity in the mRNA-protein relationship, however, results in higher levels of mRNA-protein concordance [37],[40],[41]. In prokaryotes, ribosomes bind to nascent mRNAs so that translation can be synchronous with transcription; proteins levels thus depend more directly on mRNA abundance [42]. Consistent with this, a comparison of *Staphylococcus aureus* biofilm and planktonic cells showed qualitative agreement between transcriptomic and proteomic expression differences [43], and an analysis of mRNA and protein levels for 400 genes from *Desulfovibrio vulgaris* reported correlations between 0.45–0.53 [40],[41]. In addition, genes from different functional categories display different levels of correlation. For example, in both *S. cerevisiae* and *D. vulgaris* genes associated with central intermediary and energy metabolism display higher levels of correlation than other groups [41], and a study of central metabolism genes in both wildtype *E. coli* DF11and a *pgi* mutant found a correlation of .81 between the log ratio of transcripts and the log ratio of enzyme activities [44]. Studies of transcriptional regulation of metabolism in *E. coli* [45] also suggest tight transcription-translation coupling. In unbranched segments of amino acid biosynthetic pathways, genes for enzymes catalyzing upstream reactions are transcribed earlier and with a higher promoter activity than those for downstream reactions. This pattern is optimal for rapidly producing end-products while minimizing enzyme production when enzyme levels are a direct function of mRNA levels.

Although the total amount of available enzyme sets an upper limit on the maximum flux through a particular reaction, many regulatory processes may modulate the effective levels of enzyme activity. Metabolite feedback regulation, allosteric interactions, and various covalent modifications may alter the activity of enzymes already synthesized. These modulations, however, cannot lead to more activity than is possible if all available enzyme were maximally active. For example, in the extreme case where an enzyme is completely deactivated by covalent modification (e.g. inactivation through phosphorylation), the true flux through a reaction is zero whereas mRNA levels might suggest a higher bound. With respect to E-Flux, this means that the upper bound on enzyme activity set by mRNA levels is an upper bound, but not always a tight one. In such a case, the accuracy of predictions made by E-Flux may depend on the difference between the approximate and true bounds.

It would be possible to generalize the E-Flux framework to take modulation of enzyme activity into account. In our application to mycolic acid production, we model maximum flux for a reaction as a function of all genes that are components of the corresponding enzymes or enzyme complexes. However, we could incorporate the expression of all genes whose products modulate the activity of a given enzyme into this function. For example, if an enzyme is inactivated through phosphorylation by a kinase, we may choose to model the corresponding maximum flux as a function of the expression of the genes for both the enzyme and kinase. This approach is conceptually similar to the coupling of metabolic models with Boolean regulatory models taken by Covert et al. [46], although E-Flux differs in that empirically measured gene expression levels are used. Such an approach, however, would require more knowledge of regulatory interactions between proteins than we used in the analysis presented here.

### Comparison to Previous Approaches

A number of previous methods have been developed to gain insight into metabolism from expression data. The most common method is to identify genes or sets of genes from particular pathways that are differentially expressed under different conditions [47],[48]. Often this involves visualizing expression data on metabolic maps [49]. Although useful, this approach is limited by the need for the subjective interpretation of differentially expressed gene sets, typically by an expert on the metabolic pathways of interest.

Other methods have been based on classifying gene expression by similarity to expression patterns corresponding to known metabolic or cellular states [50]–[52]. In the case of mycolic acid biosynthesis, however, different inhibitors do not cluster together based on expression similarity, either when considering all *M. tb* genes [17] or only the 29 genes directly involved with mycolic acid synthesis (Figure 5). This is consistent with the range of mechanisms by which mycolic acid biosynthesis can be suppressed. For example, isoniazid, ethionamide, and thiolactomycin inhibit the FAS-II fatty acid biosynthetic cycle, whereas cerulenin inhibits both FAS-I and FAS-II, and ethambutol blocks the incorporation of mycolic acids into the cell wall. It is possible that a gene expression-based classifier could be developed that would correctly identify inhibitors across this range of mechanisms. E-Flux, however, implicitly integrates across these different mechanisms by interpreting expression data through the lens of a metabolic network model.

More fundamentally, our method does not require a set of training data whose effect on the pathway is known in advance. Traditional classification methods require exemplars from the categories to be classified [53]. These exemplars are used to select a decision boundary in some space that places objects of one category on a different side of the boundary from objects in other categories. Although the Boshoff data set contained conditions corresponding to known mycolic acid inhibitors, this information was not used to parameterize our method. Instead, E-Flux uses a model of the underlying chemical and biological network to simulate the effects of different regulatory states. The method can thus be used to classify new expression data sets even in the absence of previous data from the same class. Moreover, we are able to predict previously unreported effects. For example, our method predicts that a small number of compounds may act to increase overall mycolic acid production although no known mycolic acid enhancers are included in the set. Furthermore, while our goal here was to predict the metabolic impact of a known external condition, in a related manuscript [54], we reverse this logic to predict an unknown environment, in particular to identify the most likely nutrient being metabolized, using expression data coupled to metabolic models.

Since the initial development of E-Flux, two other methods for combining expression data with flux balance analysis have been described. The method of Becker and Palsson [55] utilizes a variant of the method of Covert and Palsson [46] to turn genes on or off. In contrast to this approach where genes were turned off based on a Boolean model of gene regulation, the method of Becker and Palsson [55] turns off genes whose expression is below a given threshold level. If this constrained model is incapable of achieving a given objective, genes are turned back on until the objective is reachable. The method of Shlomi et al. [56] uses a novel nested optimization method to determine an FBA solution while also maximizing the correspondence between gene expression levels and metabolic fluxes. These methods differ with respect to the degree that expression data is used to modulate constrains on an FBA model. Becker and Palsson [55] apply a hard constraint using gene expression such that genes are either on or off. On the other hand, genes not turned off are not modulated by expression data. Shlomi et al. [56], in contrast, use expression data to influence fluxes indirectly, rather than through flux constraints. E-Flux falls in the middle of these two approaches. It is more aggressive than the method of Shlomi et al. [56] in that fluxes are directly constrained by expression. It is less aggressive than the method of Palsson et al. [55] in that genes are not turned off, although more comprehensive in that all flux constraints are modulated by the expression of the corresponding genes. Which approach is more accurate likely depends on the application.

### Other Applications of E-Flux

E-Flux provides a general approach for modeling metabolism from expression data. This approach has a number of potential applications, beyond the application to tuberculosis presented here. E-Flux can also be used to investigate and model other disease states where expression data are available and for which metabolic alterations are associated. For example, many cancer cells are known to grow glycolytically in the presence of oxygen and to develop a lipogenic phenotype [57]–[59]. With the availability of numerous expression data sets for various cancer cells, E-Flux may provide an opportunity to study this phenomenon computationally.

E-Flux could in principle also be used as a tool for drug discovery. For example, if a drug were sought that decreased production of a particular metabolite, then genome-scale expression profiles of a large number of small molecules could be analyzed with E-Flux and subsequent study could focus on those for which E-Flux predicted the desired inhibition. This would be a valuable approach in settings where screening directly for the effect of interest is expensive relative to microarray analysis. In addition, since E-Flux can predict unanticipated effects, the approach could be used to predict possible undesirable effects including the production of toxic metabolites. Furthermore, if a set of objective functions were developed, each corresponding to a different subsystem or pathway in the metabolic network, then E-Flux could be used separately for each objective to help identify a molecule's mechanism of action.

Finally, E-Flux provides a new tool for efforts to engineer metabolic systems. Flux analyses have been previously used to guide the design of metabolic networks. E-Flux enhances this approach by enabling the prediction of metabolic characteristics for specific empirically determined gene expression states.

## Materials and Methods

### FBA Model of Mycolic Acid Biosynthesis

We used two metabolic models: a model of the mycolic acid biosynthesis subsystem [7], and a genome-scale model for *M. tuberculosis* [6]. The small subsystem model comprises four sub-pathways: fatty acid synthase (fas) I and II, the production of malonyl-CoA which is an input for each of these, and the condensation of the products of fas I and II into alpha, methoxy and keto mycolic acids. The model has 197 distinct metabolites, linked together in 219 internal reactions. There are an additional 28 external reactions corresponding to uptake of the primary input, AccB, the free exchange of water, carbon dioxide and other substances not explicitly produced and consumed in the model, and the production of the mycolate outputs. The model is available in SBML format at DOI: 10.1371/journal.pcbi.0010046.sd001 and is presented in the supplementary material of [7]. Two genes in the model remained unknown and were labeled ‘UNK1’ and ‘UNK2’ at the time Raman et al. published the mycolic acid metabolic model. One of these was the gene or complex responsible for the dehydration of (3R)-hydroxyacyl-ACP in the fas II elongation cycle in M. tuberculosis. Subsequent to the publication of the orginal model by Raman et al., Sacco et al. [16] identified two heterodimers, Rv0635-Rv0636 (HadAB) and Rv0636-Rv0637 (HadBC) which perform this role. They observed substrate specificity for these dimers, with hadAB preferentially catalyzing this reaction for shorter carbon chains and hadBC doing so for longer carbon chains. We included catalysis of reactions 68 and 74 (length up to C-18) by hadAB, and reactions 80, 86, 92, … ,188 (longer lengths) by hadBC. Our results were not substantially altered by including the hadABC genes.

The genome-scale model we used was closely based on that published by Beste et al. [6]. We merged Raman et al.'s mycolic acid submodel [7] into the genome-scale model so that we could use both models with E-Flux to test for inhibition or enhancement of mycolic acid production capacity. The merging was done as follows: we identified all external metabolites of Raman et al.'s model and found the equivalent metabolite in Beste et al.'s model. We then removed exchange reactions for these metabolites so that net production and consumption of these was no longer allowed. We removed mycolic acid reactions from the genome-scale model and replaced them with the mycolic acid reactions in Raman et al.'s model, and normalized the bounds on exchange reactions so these were uniform (+/−1). The net result was to replace McFadden *et al*.'s representation of mycolic acids with that of Raman et al., as the latter is more detailed and as this allows direct comparison of the results of E-Flux in the two models. The model is available as Dataset S1.

### Expression Data

The expression data published by Boshoff et al. [17] are listed under GEO accession number GSE1642. Boshoff et al. used clustering of the expression profiles to predict the mechanisms of action of previously unknown agents. Data are available for two channels: Cy3 (control) and Cy5 (condition) on a total of 437 spotted chips, each with mRNA expression data for M. tuberculosis strain H37Rv. The published data are in log format; these were exponentiated to obtain raw values. The data of Karakousis et al. [18] are published at http://www.ncbi.nlm.nih.gov/geo/ under accession number GSE9776 and are also two-channel data of H37Rv *M. tb*; the dataset contains 17 arrays for 6 unique conditions, comparing *M. tb*'s response to isoniazid in dormancy models.

### Expression Data Processing

We processed the expression data using MAANOVA 2.0 [20], a Matlab package for analyzing data from two-dye cDNA microarray experiments. MAANOVA 2.0 fits an analysis of variance (ANOVA) model to the data to account for non-biological variation in the measurements. Briefly, let *y_ijkg_* denote the log-transformed measurement from the *i*th channel, *j*th chip, *k*th variety (experimental condition), and *g*th gene. Then we fit the model
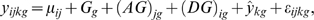
where *μ_ij_* is the average measurement for channel *j* of chip *i*, *G_g_* represents the effect of gene *g*, *(AG)_jg_* represents effects specific to chip *j* and gene *g*, *(DG)_ig_* represents effects specific to channel *i* and gene *g*, 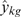 represents effects specific to variety *k* and gene *g* (i.e. the biological variation), and *ε_ijkg_* is error. Thus we fit for, and subtract out, systematic, non-biological effects such as overall brightness and spot effects. We fit the model such as to minimize the residual sum of squares (RSS) given by
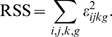
The estimate of 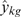 given by this procedure is used as the ANOVA-processed data.

We compared the results of our method using ANOVA-processed data with those using the published values without statistical filtering. In this approach, the published data (log format) were exponentiated to obtain raw values. To remove noise resulting from variation in overall brightness from chip to chip while preserving median differences between condition and control channels, we adjusted the medians of each chip according to the median of that chip's Cy3 channel. We set the median of all control channels to the maximum of the control channel medians across the dataset (rather than a middle value) to avoid obtaining negative flux constraint inputs. In other words, we computed the median of each chip's Cy3 channel (denoted M_j_ for the j'th chip), and found the maximum of these, M_max_. We then added (M_max_-M_j_) to both channels of the j'th chip, for each j. The resulting values had the same median for the Cy3 channels, and different medians for the Cy5 channels, while the difference between Cy3 and Cy5 channel medians on each chip was the same as in the raw data. We also performed E-Flux using log-transformed values; predictions using log-transformed and raw values were correlated with R = 0.99 after the control-channel median adjustment described here.

Comparing predictions based on ANOVA-processed and raw expression data (with median adjustment), our results for the top mycolic acid inhibitors (isoniazid, thiolactomycin, ethionamide) are preserved, as are the inhibitory predictions for cerulenin, PA-824 and valinomycin. Ethambutal was also a predicted inhibitor from the unprocessed data, but not so strongly, along with streptomycin. The enhancing effect of triclosan was more strongly predicted from the raw data than the ANOVA-processed data. ZnSO4 was inhibitory but not as strongly, and GSNO was not as strongly enhancing. Overall, results from the two data sets were positively correlated with R = 0.62 at a significance level p = 2.11e-48.

For the data on isoniazid and dormancy from Karakousis et al. [18], the ANOVA model had fewer data points in total (17 microarrays) so the corresponding error bars (see Figure 4) are wider than for the Boshoff data reflecting a greater residual sum of squares.

### E-Flux Method

The E-Flux method consists of creating constraint vectors *a* and *b* from expression data for control and condition channels, using these as constraints for flux balance analysis with a given objective, and comparing the maximum production capacity of that objective between the control and condition.

We constructed constraint vectors *a* and *b* for the control and condition channels for each condition in the compendium of expression data as follows. For each reaction that is catalyzed by only one gene, we set the upper bound *b*_j_ to the expression value for the gene whose product catalyzes reaction *j*, taking the value from the relevant data. For example, reaction 2 in the mycolic acid sub-model is catalyzed by gene Rv3279c corresponding to birA [7], so if the control channel expression value for Rv3279c is 15, *b_2_* is initially set to 15. For each reaction catalyzed by a complex requiring two genes we set *b*_j_ to the minimum of the expression of the two genes, and for reactions which can be catalyzed by either gene we set *b*_j_ to the sum of their expression values. We then normalized the *b*_j_ values to 1 by dividing each component of *b*_j_ by M = max_j_ (*b*_j_). For each exchange reaction, we set *a*_j_ = −1 and *b*_j_ = 1; in other words, these reactions were not constrained by gene expression. Changing the constraint (for example, from +/−1 to +/−2) on exchange reactions to another value does not change the results presented here, as the relative production capacity from control to condition is a log ratio and is not dependent on the overall scale. Following Raman et al., all internal reactions in the model were modeled to be irreversible, so that *a*_j_ = 0 for these (*j* = 1 to 219). For the reactions catalyzed by the remaining unknown gene in Raman's model we set *b*_j_ = 1 and *a*_j_ = 0.

All of these steps were performed for the control channel expression data and then separately for the condition channel. This yields 4 vectors: *a*_control_ and *b*_control_ taken from the cy3 channel of the chip, and *a*_condition_ and *b*_condition_ taken from the condition channel. Linear optimization was then performed with each set of constraints and the same objective function, namely a weighted production of mycolic acids. The objective function for the mycolic acid subsystem model was
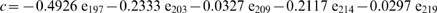
where e_i_ represents the vector (0, 0, …, 1, …, 0) with a 1 in the i'th component and 0 in all other components. Since the linear programming tool linprog in matlab *minimizes* c^T^v, the coefficients of *c* were chosen to be negative so that the optimization *maximizes* (−c^T^v), namely the weighted flux creating mycolic acids. The reactions included in *c* produce alpha, cis- and trans- methoxy mycolate and cis- and trans- keto mycolate respectively. This is the same as objective function *c*_1_ in Raman et al. Our results are insensitive to the particular balance between the α-, keto- and methoxy- mycolates in the objective function. In the genome-scale model our objective function was the same but the reactions are numbered differently. We also performed E-Flux with two alternative objective functions: biomass as given in Beste et al., and the mycolic acid objective function with the same weights as given in [7].

This procedure, with either objective function, yields two results for the maximal production capacity: P_control_ = max(c^T^v) using constraints taken from the control channel, and P_condition_ = max(c^T^v) using constraints taken from the condition channel. The relative production, namely the results shown in Figure 3 and Supplementary Figures S1, S2, S3, S4 and S5, is given by log( P_condition_/P_control_).

In addition to setting *b*_j_ equal to the expression value for the gene catalyzing the j'th reaction, we explored using sigmoidal, exponential and polynomial increasing functions to create the constraint *b*, so that rather than b_j_ = expression(*j*), we used b_j_ = *f*(expression(*j*)) where *f* is sigmoidal, exponential or polynomial. The results presented in Table 1 are robust to saturation at high expression levels and suppression at low levels, as long as these retain sufficient variation in the data.

### Significance Calculations and Error Bars

To determine whether predictions were significant we resampled the control channel of each chip, adding noise sampled from the noise distribution given by the Anova model as described above. We compared the mycolic acid production capacity from one such resampled control dataset to another, and repeated the procedure 800 times. We then found the “null” interval in which 95% of the values lie. The interval is denoted by the dotted lines in Figure 3. Predictions lying within this interval were not considered significant. To generate the error bars shown in Figure 3, we used a similar approach. Here, we added noise (again distributed in accordance with the anova model) to both the control and condition channel of the chip, and applied E-Flux. After repeating this procedure 800 times, we found the intervals in which 95% of the values lay; these form the error bars shown. They represent the uncertainty in our predictions based on the Anova estimate of how much random noise there is in the data.

### Mycolic Acid Specificity Calculation

To make a distinction between predictions that are specific to the mycolic acid pathway and those that may result from the conditions' effects on a large number of genes in M. tuberculosis, we used two approaches. For the first, we computed predictions using randomly chosen genes from each chip in place of the genes in the metabolic model. To do this, we chose a random permutation of all genes on each chip, and these randomly chosen genes (rather than the genes actually listed in the metabolic model) to form the constraint vectors a and b. This was done for both control and condition channels of each chip, using the same gene permutation for the two channels. We then applied E-Flux to recompute the predicted inhibition or enhancement using constraints from expression of the randomly selected genes. After repeating this procedure, we found the range in which 95% of the resulting predictions lie, for each experiment (grey bars in Figure 3 and Supplementary Figures). Where the range is large, there is typically substantial overlap between the gene relabelled predictions and the predictions using the correct genes for the metabolic model. This results from the condition having affected many genes in the organism. In this case we do not consider the prediction to be specific to the mycolic acid pathway because there may be inhibition or enhancement of a number of pathways in the organism, leading to a likely inhibition when in the model when random genes are used. Alternatively, where there is considerable difference between the gene-relabelled predictions and the noise-resampled predictions from the correct genes, as is the case with isoniazid (see Figure 3), the predictions of E-Flux are considered specific to mycolic acids. Our quantitative use of “specific” required that 95% of the noise-resampled predictions (i.e. those which give the error bars shown in Figure 3) lie outside the 95% range of the gene-relabelled predictions (grey bars in Figure 3). By this criterion, when a prediction is deemed “specific” it is unlikely that that prediction would be obtained with randomly relabelled genes.

## Supporting Information

Figure S1 Predictions of E-flux applied to mycolic acid biosynthesis in M. tuberculosis. First set in alphabetical order from the Boshoff expression data compendium.
(3.16 MB TIF)

Figure S2 Predictions of E-flux applied to mycolic acid biosynthesis in M. tuberculosis. Second set in alphabetical order from the Boshoff expression data compendium.
(3.16 MB TIF)

Figure S3 Predictions of E-flux applied to mycolic acid biosynthesis in M. tuberculosis. Third set in alphabetical order from the Boshoff expression data compendium.
(3.16 MB TIF)

Figure S4 Predictions of E-flux applied to mycolic acid biosynthesis in M. tuberculosis. Fourth set in alphabetical order from the Boshoff expression data compendium.
(3.16 MB TIF)

Figure S5 Predictions of E-flux applied to mycolic acid biosynthesis in M. tuberculosis. Fifth set in alphabetical order from the Boshoff expression data compendium.
(3.16 MB TIF)

Dataset S1 Integrated genome-scale model in XML format
(1.13 MB XML)
