# Supplementary figures and images for: Interpreting Expression Data with Metabolic Flux Models: Predicting Mycobacterium tuberculosis Mycolic Acid Production
Source: PLoS Comput Biol. 2009 Aug 28;5(8):e1000489. doi: 10.1371/journal.pcbi.1000489 (PMC2726785; doi:10.1371/journal.pcbi.1000489)

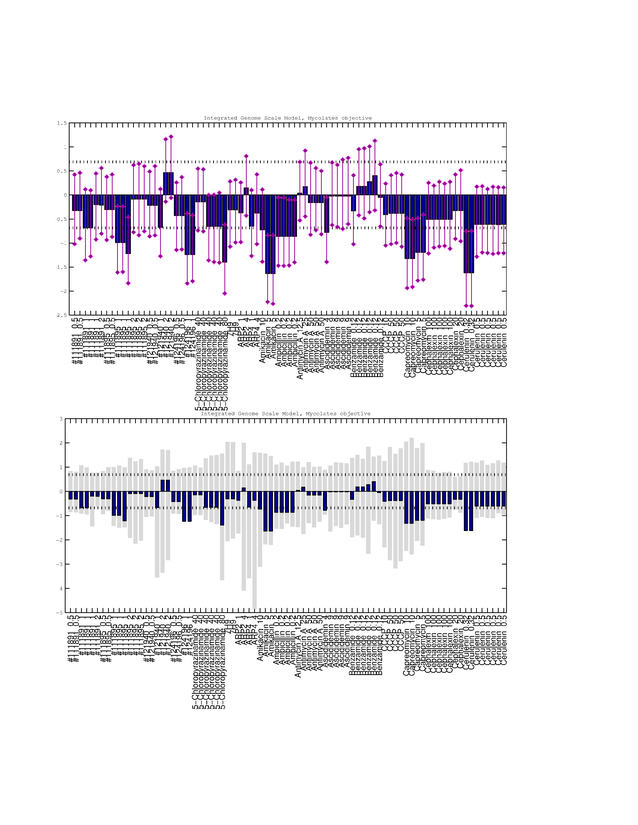

Supplement: Figure S1 — Predictions of E-flux applied to mycolic acid biosynthesis in M. tuberculosis. First set in alphabetical order from the Boshoff expression data compendium. (3.16 MB TIF) [file pcbi.1000489.s001.tif]

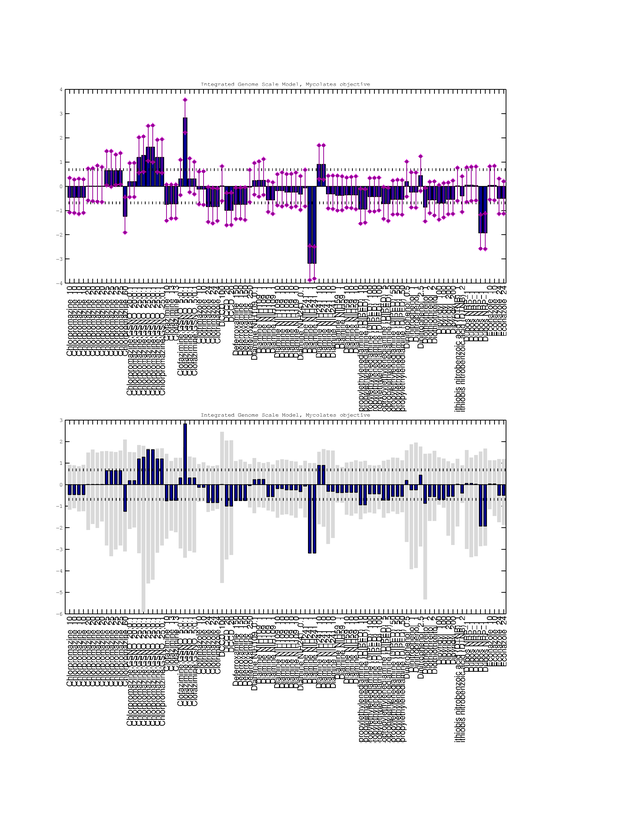

Supplement: Figure S2 — Predictions of E-flux applied to mycolic acid biosynthesis in M. tuberculosis. Second set in alphabetical order from the Boshoff expression data compendium. (3.16 MB TIF) [file pcbi.1000489.s002.tif]

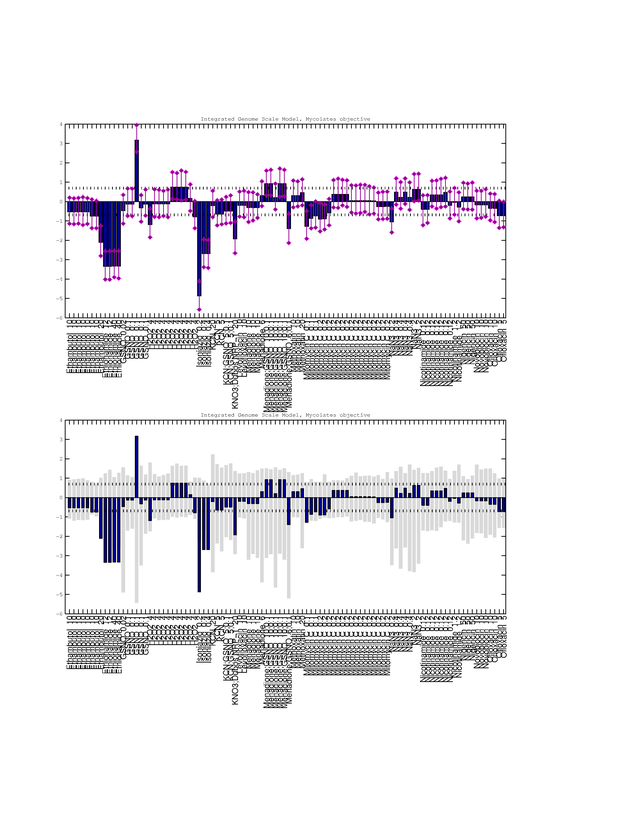

Supplement: Figure S3 — Predictions of E-flux applied to mycolic acid biosynthesis in M. tuberculosis. Third set in alphabetical order from the Boshoff expression data compendium. (3.16 MB TIF) [file pcbi.1000489.s003.tif]

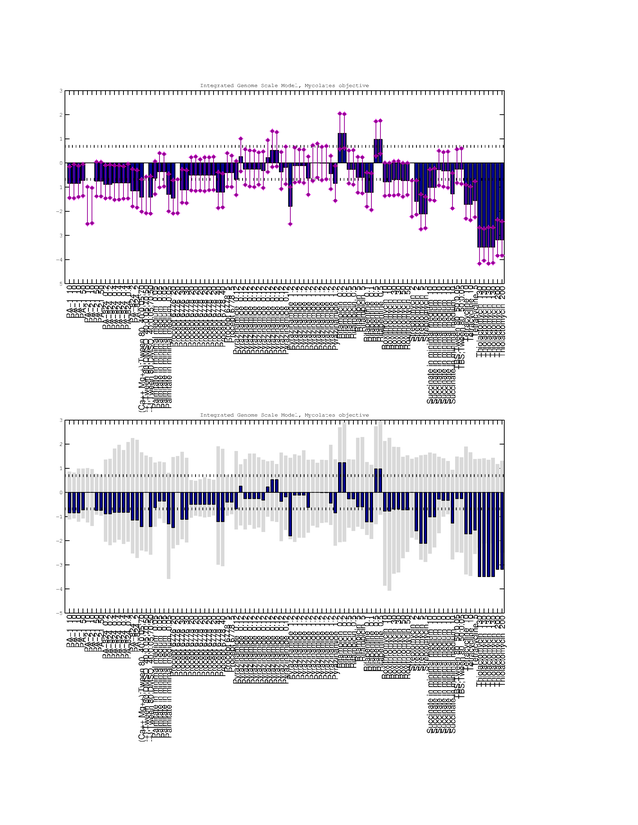

Supplement: Figure S4 — Predictions of E-flux applied to mycolic acid biosynthesis in M. tuberculosis. Fourth set in alphabetical order from the Boshoff expression data compendium. (3.16 MB TIF) [file pcbi.1000489.s004.tif]

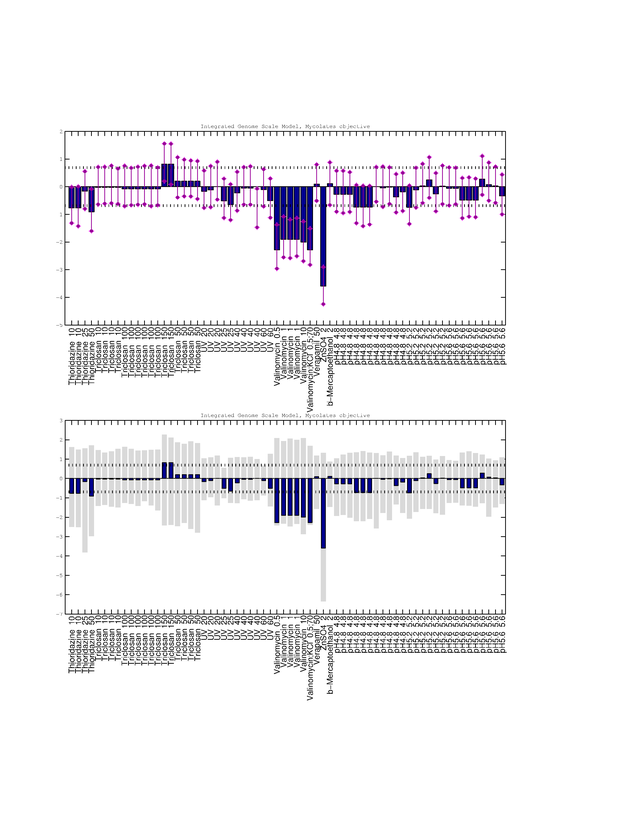

Supplement: Figure S5 — Predictions of E-flux applied to mycolic acid biosynthesis in M. tuberculosis. Fifth set in alphabetical order from the Boshoff expression data compendium. (3.16 MB TIF) [file pcbi.1000489.s005.tif]
